# Innovation with a Footnote: A Rare Limitation in an Otherwise Promising Endograft

**DOI:** 10.1016/j.ejvsvf.2026.01.006

**Published:** 2026-01-31

**Authors:** Nikolaos Kontopodis, Christos V. Ioannou

**Affiliations:** Vascular Surgery Department, University Hospital of Heraklion, University of Crete Medical School, Heraklion, Crete, Greece

Endovascular aneurysm repair is the preferred treatment modality for abdominal aortic aneurysms.^1^ Various endoluminal systems are currently available and each has its own advantages and limitations. While there are straightforward cases that could be treated with most of the available endografts, there are certain anatomic conditions that render one system more appropriate, or inappropriate, than the other.^2^

The ALTO (Endologix, Irvine, CA, USA) endograft introduced a unique sealing concept.^3^ The sealing and fixation modes are separated, allowing for an ultra low profile delivery system, enhancing the ability to navigate through narrow access vessels. Sealing is not achieved with central stents but with two polymer filled O rings, with the top ring creating a conforming seal, to treat aortic necks ≥7 mm in length (shortest neck length within graft instructions for use [IFU]). These are filled through integrated tubes that begin in the ipsilateral limb port and connect to three and a half rings in the ipsilateral leg, two proximal O rings, and three and a half rings on the contralateral leg. The main body is otherwise unsupported, with its deployment being completely dependent on successful polymer filling. These characteristics allow for a greater proportion of anatomies to be treated on label compared with the competition.^4^

This unique design may rarely cause problems in specific conditions. In this issue of *European Journal of Vascular and Endovascular Surgery Vascular Forum*, using an experimental design, Yokoyama et al. suggest that in cases with a high infrarenal angulation (90°), the polymer filled channels may malfunction, resulting in failure of polymer insertion into the rings, which is especially true when the ipsilateral limb is compressed against the lesser curve of the aortic neck.^5^ Although the experimental design may not be realistic in all cases (i.e., nosecone facing downwards), according to our experience, a polymer filling malfunction may occur in real life. Having removed the stiff guidewire (suggested in the IFU), extreme angulation may compress the port, inhibiting satisfactory polymer ring filling with the autoinjector. The authors’ conclusion provides a useful tip in such cases, which is to leave the stiff guidewire in place when the polymer is connected to the device, since its straightening effect could better facilitate polymer filling. Additionally, they propose deploying the endograft so that the ipsilateral limb is on the greater and not the lesser curve of the aortic neck, to reduce compression on the port. This is an easy manoeuvre that does not increase technical difficulty or procedure time.

Our team has also encountered polymer filling difficulties in two (out of 396 Ovation/Alto) cases and has found some additional useful manipulations to overcome such problems. Firstly, one can push the delivery system slightly upwards, after straightening the system by re-inserting the stiff wire and then manually pushing the polymer by hand in a pulsatile fashion (to unkink the channel). After it starts to fill, the auto injector is connected, allowing the system to reach the manufacturer's suggested pressure (0.8 atm). Secondly, by using the integrated crossover wire, traction to both limbs is exerted, which straightens the graft facilitating polymer filling (Fig. 1). Finally, inserting the endograft in an anteroposterior (AP) fashion will displace the polymer port from the aortic wall, therefore rendering this configuration beneficial in such extreme cases. The authors' approach (ipsilateral limb on the greater curve) may indeed decompress the port, allowing polymer fill of the ipsilateral leg and proximal rings, but in this case the contralateral limb's channels may compress, resulting in its failure to expand. AP deployment could prove beneficial in this scenario. Notably, infrarenal angulation is not reported in the IFU of the device, so this limitation may not have been recognised by the manufacturer. The fact that this kind of malfunction has not been reported in large administrative endograft databases is indicative of its rarity, which may indeed happen in extreme infrarenal angulations.

**Figure 1:**
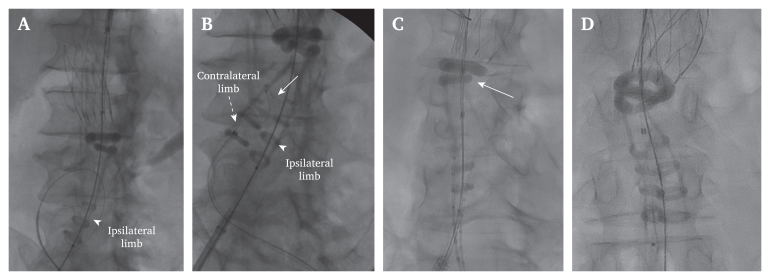
Intraoperative angiographies of two cases in which the polymer did not fill the contralateral leg ring that resolved using the integrated crossover wire, resulting in its successful expansion. (A) Note the infrarenal angulation; even though it was <45°, kinking of the second sealing ring was observed. The graft was inserted in a ballerina orientation to facilitate easier contralateral limb catheterisation, but the polymer was obstructed from running down towards the contralateral limb due to compression. Since the contralateral limb did not fill with polymer, it was not visualised and thus could not be catheterised. (B) A crossover 0.014” wire (solid white arrow) was used to facilitate limb catheterisation. At that point, the polymer started to flow into the contralateral limb (dotted white arrow), the limb was catheterised, and the procedure was successfully completed. (C) A case without significant angulation (white arrow), in which the contralateral limb channel did not fill at the level of the first half ring. (D) Again, placement of the crossover 0.014” wire caused the polymer to start flowing. A 0.035” wire was easily passed up in parallel via the lasso sheath and after securing a stiff wire in the contralateral limb, the 0.014” crossover wire and sheath were removed and the procedure proceeded as usual.

A few anecdotal cases have been reported.^6^^,^^7^ A Japanese Committee report with 1 116 ALTO implantations describes 12 cases with unsuccessful polymer fill, with proximal O ring filling failure in four (0.36%) and contralateral limb ring filling failure in eight cases (0.72%).^8^ We have previously published another case of contralateral limb ring filling failure followed by limb collapse, not attributed to aortic angulation, but to a very narrow distal aortic lumen, which caused a similar compression effect.^9^

In conclusion, the benefits of the ALTO system come with the limitation if its unsupported main body, which under specific and extreme anatomic conditions may result in unsuccessful and incomplete polymer filling and failure to deploy the device. Failure to deploy may result in serious clinical sequalae, including the need for open conversion and endograft explantation. Awareness of these situations and the use of these simple measures can ensure normal graft sealing ring polymer filling, ensuring adequate aneurysm sac exclusion.

## Funding

This research did not receive any specific grant from funding agencies in the public, commercial, or not for profit sectors.
